# Overuse of non-prescription analgesics by dental clinic patients

**DOI:** 10.1186/1472-6831-8-33

**Published:** 2008-12-09

**Authors:** Kennon J Heard, Nicole L Ries, Richard C Dart, Gregory M Bogdan, Richard D Zallen, Frank Daly

**Affiliations:** 1 Rocky Mountain Poison and Drug Center, Denver Health, Denver Health Dental Clinic, Denver Health, University of Colorado Denver School of Medicine Division of Emergency Medicine, Denver, CO, USA; 2 The Department of Emergency Medicine, Royal Perth Hospital and The University of Western Australia, Australia

## Abstract

**Background:**

Many patients present to dental clinics for treatment of painful conditions. Prior to seeking treatment, many of these patients will self-medicate with non-prescription analgesics (NPA), and some will unintentionally overdose on these products. The objective of this study is to describe the use of NPA among dental patients.

**Methods:**

All adult patients presenting to an urban dental clinic during a two-week period in January and February of 2001 were approached to participate in this research project. Trained research assistants using a standardized questionnaire interviewed patients. Patient demographics and the NPA usage over the 3 days preceding the office visit were recorded. We defined a supra-therapeutic dose as any dose greater than the total recommended daily dose stated on package labeling.

**Results:**

We approached 194 patients and 127 participated. The mean age of participants was 35.5 years, 52% were male. Analgesic use preceding the visit was reported by 99 of 127 patients, and most (81/99) used a NPA exclusively. Fifty-four percent of NPA users were taking more than one NPA. NPA users reported using ibuprofen (37%), acetaminophen (27%), acetaminophen/aspirin combination product (8%), naproxen (8%), and aspirin (4%). Sixteen patients reported supra-therapeutic use of one or more NPA (some ingested multiple products): ibuprofen (14), acetaminophen (3), and naproxen (5).

**Conclusion:**

NPA use was common in patients presenting to a dental clinic. A significant minority of patients reported excessive dosing of NPA. Ibuprofen was the most frequently misused product, followed by naproxen and acetaminophen. Though mostly aware of the potential toxicity of NPA, many patients used supra-therapeutic dosages.

## Background

Pain is a common complaint among patients presenting for emergency care at a dental clinic. Non-prescription analgesic pain relievers are commonly used by patients with dental pain. They decrease pain when administered after dental extractions [1-3] and when administered prior to dental surgery [4]. There are fewer studies evaluating the utility of these products for dental pain not related to dental procedures. Korberly studied patients presenting to a dental clinic for acute pain and found that both acetaminophen (1 g) and aspirin (1 g) decreased pain relative to placebo.[5] These studies all suggest that non-prescription analgesics are an effective therapy for dental pain.

When the manufacturer's recommended dosing is followed, these medications are very safe. Still, two recent reports have highlighted the risk of accidental acetaminophen overdose during treatment of dental pain[6,7] Patients with occult acetaminophen overdose may present dramatically with acute liver failure, and there are risks from over use of non-steroidal anti-inflammatory drugs (NSAIDS). While doses of ibuprofen under 1200 mg/day minimally increase the risk of gastrointestinal (GI) bleeding (relative risk 1.1 vs no medication), the prescription dose increases the risk of bleeding dramatically (relative risk of 4 vs no medication).[8] The risk is higher with prolonged use, but one study has reported patients starting naproxen are at higher risk than those starting ibuprofen and that that difference is detectable within 14 days.[9] This suggests that even a few days of use results in increased potential for injury. As some estimate that up to 15,000 people die per year in the United States as a complication of their NSAID treatment [10], NSAID overuse is a potential major health issue.

Given the severity of dental pain and the difficulty that many patients have accessing dental care, we believe that patients presenting to a dental clinic may represent a group at high risk of unintentional overdose. The purpose of this study was to characterize the use of non-prescription analgesics by patients at an urban dental health clinic.

## Methods

All adult patients presenting to an urban dental clinic provided at a safety net hospital during a two-week period were approached to participate in the research project. This study was conducted in January and February of 2001. Patients of either gender, 18 years of age or greater were included if they provided informed consent. We excluded patients who did not speak English or who were unwilling or unable to provide informed consent. The local Institutional Review Board approved this study.

The clinic is open from 08:00 to 17:00 with resident coverage overnight and provides care for 17,000 patient encounters per year. The primary patient complaint at these encounters is dental pain. Trained research assistants using a structured questionnaire approached all patients in the clinic. During the survey, patients were asked to name which non-prescription analgesic products they had used (including doses) on the day of the visit and in the 2 days preceding their visit. A book of photographs displaying the packaging for the 22 most popular analgesic products was used as a prompt when needed. Patients who gave a history of supra-therapeutic use of a non-prescription analgesic were given a printed sheet advising them to seek medical advice in case they experienced abdominal pain, nausea, vomiting, or yellowing of the skin. No attempt was made to assess the patient for signs of toxicity.

After the data collection was complete, responses were tabulated and entered into Microsoft Access. When the patient provided a specific dose, this dose was accepted and entered as the dose. When a patient did not know the dose, the interviewer attempted to obtain an exact product name and the amount taken. When an exact product name was provided, the dose of each medication in the product was determined from a standard database (Micromedex^®^, Englewood CO), if the patient could recall the number of pills ingested. If an exact product could not be determined, the lowest dose formulation of the medication available was used to determine the dose ingested by the patient. The maximum daily non-prescription doses were determined from the manufacturer's label and are shown in Table 1. Patients reported daily doses in excess of these values were counted as overusers. Proportions and percentages (with 95% confidence intervals) were reported for all responses. Relative risks with 95% confidence intervals were used to compare sex, age group and type of analgesic used between overusers and non-overusers.

**Table 1:** Maximum daily non prescription and prescription doses for acetaminophen, aspirin, ibuprofen and naproxen

**Medication**	**Non-prescription**	**Prescription**
Acetaminophen	4000 mg	4000 mg
Aspirin	3900 mg	3900 mg^1^
Ibuprofen	1200 mg	2400 mg
Naproxen sodium^2^	660 mg	1100 mg

1. For rheumatoid arthritis the dose may be increased to achieve a serum concentration of 30 mg/dl.

2. To convert to naproxen decrease the dose by 10%.

## Results

We approached 194 patients, of which 127 participated. Of the 67 patients who did not participate, 37 were not interested or in too much pain to participate and 30 did not speak English. The participants had a mean age of 35.5 years (range 18 to 68 years) and had a slight male predominance (52%). Non-participants were of similar age and gender. Overall, 99/127 (78%; 95% confidence interval [CI] 70 to 84%) patients reported using an analgesic. Eighteen (14%; CI 8 to 21%) patients were taking a prescription analgesic only, 37 (29%; CI 21 to 38%) were taking a single non-prescription analgesic. Twenty-nine (23%; CI 15 to 21%) patients were taking two medications, 12 (9%; CI 5 to 16%) patients were taking three medications, and 3 (2%) patients were taking 4 medications.

Acetaminophen, as an non-prescription preparation, was used by 34/127 patients (27%; CI 19 to 35%), non-prescription ibuprofen was used by 47 patients (37%; CI 28 to 46%), acetaminophen/aspirin combination products were used by 10 patients (8%; CI 3 to14%), non-prescription naproxen was used by 10 patients (8%; CI 3 to 14%), and aspirin was used by 6 patients (4%; CI 2 to 10%). Twenty-six patients (26%; 18 to 36%) were taking a prescription medication that contained acetaminophen as an ingredient in addition to at least on non-prescription analgesic, including 3 who took non-prescription acetaminophen.

Sixteen percent of non-prescription analgesic users reported exceeding the manufacturer’s recommended daily dosage. The median doses, interquartile ranges and ranges for patients who reported exceeding the recommended dose is shown in table 2. The incidence of overuse for each medication was ibuprofen 14/47 (23%; CI 12 to 38%); acetaminophen 3/34 (12%; CI 3 to 27%); and naproxen 5/10 (50%; CI 19 to 81%). Thirteen patients exceeded the recommended dose for a single non-prescription analgesic (4 naproxen and 9 ibuprofen), while three reported exceeding the recommended dose for ibuprofen and acetaminophen and one exceeded the recommended dose for ibuprofen, acetaminophen and naproxen. No patient exceeded the recommended dose of aspirin or of acetaminophen/aspirin combination products. Three patients were taking acetaminophen in addition to a prescription combination analgesic medication containing acetaminophen. As we only gathered dose data on non-prescription analgesic, we could not determine if the combined total acetaminophen dose of the non-prescription analgesic product and prescription product exceeded the maximum recommended dose of acetaminophen in these three patients.

**Table 2:** Maximum daily dose (mg) for patients who exceeded the recommended non-prescription dose of analgesics.

**Medication**	**N**	**Median**	**Interquartile range**	**Range**
Acetaminophen	3	6000	5000–9000	5000–9000
Ibuprofen	14	1600	1600–3500	1600–12000
Naproxen sodium	5	880	880–2035	880–2200

The risk of overuse by men was 1.3 the risk for women (95% CI 0.5 to 3.3). The rate of overdose for patients age <30 years, 31–50 years, and >50 years were 5/50 (10%; 3 to 22%), 11/59 (19%; 10 to 31%) and 0/17 (0; 0 to 19%) respectively. These rates were not significantly different. Ibuprofen and naproxen users were more likely [RR = 1.6 (95% CI 0.55 to 4.9) and 4.2 (95% CI 1.4 to 12.9) respectively] to overuse than acetaminophen users.

## Discussion

The vast majority of dental clinic patients interviewed were using a product containing a non-prescription analgesic. The pattern of non-prescription analgesic use has been changing as newer products become available. Studies in the 1970s reported that dental clinic patients primarily used aspirin [11], while studies in the 1980s reported a majority of patients using acetaminophen [12]. It now appears that ibuprofen is the medication of choice for the majority of patients, although acetaminophen remains a common choice. This is similar to the medication selection pattern reported for emergency department patients [13].

A substantial minority of patients exceeded the maximum daily dosage recommended by the manufacturer. The rate of overuse is similar to the rate reported by Preshaw 25 years ago. In that study, 7.6% of patients in a British dental practice reported excessive dosing [14]. This suggests that the issues that lead to misuse have existed for many years.

The reason for overuse cannot be determined from the present study design. We suspect it is multi-factorial. One issue is the inadequate use of preventative dental care that allows caries to progress to the point where they invade the pulp and cause pain. This may be due to cost, lack of an available provider or patient apathy. Regardless, these issues are beyond the scope of our research. Once pain occurs, patients may become desperate and can resort to extreme measures to obtain relief. One way patients seek relief is by taking prescription medications (obtained from old prescriptions, friends or purchased on the black market). This may have several health implications. We did not attempt to quantify acetaminophen use in prescription products, but accidental acetaminophen overdose due to overuse of acetaminophen/opioid combination products is well described.[15] Simultaneous ingestion of prescription and nonprescription acetaminophen products is another cause of inadvertent overdose. We identified several cases of simultaneous prescription and non-prescription use, suggesting that this is a fairly common event. Further work is required to better characterize this issue.

The frequent overuse of non-prescription analgesics has several safety implications. These medications are safe when used in recommended doses. However, the adverse effects of these medications are clearly dose-related, and our data suggest that patients are exceeding the recommended doses. The continued non-prescription availability of these medications requires that the risk/benefit analysis consider some patients will not use these medications as directed, and therefore be subject to risk above those associated with non-prescription dosing for NSAIDS and will also result in patients who develop liver injury or failure from accidental acetaminophen overdose.

The second implication is that patients lack adequate education regarding the appropriate use of non-prescription analgesics. Cham et al, conducted a survey with patients presenting to an emergency department to determine the prevalence of non-prescription analgesic use and measure patient knowledge about the potential adverse effects of misuse of these products. This study found an incidence of non-prescription analgesic use similar to the one reported in our study (67%; 143/213 reported NSAID use and 60%; 127/213 reported use of acetaminophen). Cham also found that many, 64% of the patients surveyed, were ill informed about the potential adverse effects from misuse of non-prescription analgesic preparations [16]. One plausible explanation for these findings is that while patients have a general idea that "too much is bad", they do not know the appropriate doses of these medications and the specific adverse reactions that occur when these doses are exceeded. If this explanation is correct, then there is a need for improved patient education.

Healthcare providers have a clear opportunity for intervention. Ideally, this intervention would be education and prevention. Counseling patients on appropriate treatment of dental pain could be a part of routine dental care. However, as many patients do not present to dental care until symptoms have developed, there may be no opportunity for prevention in many cases. In these cases, it is important that dentists ascertain the extent of non-prescription analgesics use to identify patients who have taken potentially dangerous doses of these medications. Once these patients are identified, the provider should provide education about appropriate non-prescription analgesic use, and possibly refer these patients for evaluation for occult toxicity from these drugs.

## Limitations

These results indicate there is potential for supra-therapeutic dosing in patients who are experiencing dental pain. However, the results of this survey are from a small sampling of dental patients and only over a 2-month timeframe. In addition, patient recall of non-prescription analgesic use over the past 72 hours may have been incomplete or inaccurate. We attempted to improve product identification by providing patients with a book of photographs with the 22 most popular analgesics to help recall the type of medication used. There may have been other sources of acetaminophen or non-steroidal anti-inflammatory medications in cold and cough preparations, for example. Our study did not measure the duration of use, which can also alter the risk for toxicity. Finally, we only measured the prevalence of overuse and did not determine if any patients had injury from overuse.

## Conclusion

Non-prescription analgesic use is common in patients presenting to a safety net dental clinic, and a significant minority of these patients overuse these products. Nonsteroidal anti-inflammatory drugs are the most frequently misused products, but patients also overuse acetaminophen. This overuse may place patients at increased risk for adverse events from these products.

## Competing interests

Drs Heard, Reis, Bogdan, and Dart were employed by the Rocky Mountain Poison and Drug Center while working on this manuscript. The Rocky Mountain Poison and Drug Center has research, consulting and clinical contracts with McNeil Consumer Healthcare, a manufacturer of acetaminophen and ibuprofen products. The authors received only their salary for work performed under these contracts. The authors have no other competing interests.

## Authors' contributions

RD, GB, RZ, FD and KH designed the study. GB and FD were responsible for data colleciton. NR and KH analyzed the data. NR and KH wrote the first draft and all authors contributed substantially to the revisions. KH takes responsibility for the paper as a whole.

## Pre-publication history

The pre-publication history for this paper can be accessed here:


